# A questions-based investigation of consumer mental-health information

**DOI:** 10.7717/peerj.867

**Published:** 2015-03-31

**Authors:** Colleen E. Crangle, Joyce Brothers Kart

**Affiliations:** 1 Converspeech LLC, Palo Alto, CA, USA; 2 Psychotherapist (LMFT) in Private Practice, Palo Alto, CA, USA

**Keywords:** Mental health, Information, Consumer health informatics, Consumer questions, Crowdsourcing, Question analysis

## Abstract

Despite the wealth of mental-health information available online to consumers, research has shown that the mental-health information needs of consumers are not being met. This study contributes to that research by soliciting consumer questions directly, categorizing them, analyzing their form, and assessing the extent to which they can be answered from a trusted and vetted source of online information, namely the website of the US National Institute of Mental Health (NIMH). As an alternative to surveys and analyses of online activity, this study shows how consumer questions provide new insight into what consumers do not know and how they express their information needs. The study crowdsourced 100 consumer questions through Amazon Inc.’s Mechanical Turk. Categorization of the questions shows broad agreement with earlier studies in terms of the content of consumer questions. It also suggests that consumers’ grasp of mental health issues may be low compared to other health topics. The majority of the questions (74%) were simple in form, with the remainder being multi-part, multifaceted or narrative. Even simple-form questions could, however, have complex interpretations. Fifty four questions were submitted to the search box at the NIMH website. For 32 questions, no answer could be found in the top one to three documents returned. Inadequacies in the search and retrieval technology deployed at websites account for some of the failure to find answers. The nature of consumer questions in mental health also plays a role. A question that has a false presupposition is less likely to have an answer in trusted and vetted sources of information. Consumer questions are also expressed with a degree of specificity that makes the retrieval of relevant information difficult. The significance of this study is that it shows what an analysis of consumer mental-health questions can tell us about consumer information needs and it provides new insight into the difficulties facing consumers looking for answers to their questions in online resources.

## Background

There is a wealth of vetted mental-health information specifically for consumers ^1^1The term *consumer* refers to anyone who uses health care services or might use health care services. available online from trusted sources such as the US National Institute of Mental Health (NIMH) and SAMHSA (the US Substance Abuse and Mental Health Services Administration). Trusted community organizations such as NAMI (the US National Alliance on Mental Illness) also provide comprehensive vetted information on mental health for consumers. Many other countries have similar online resources—the UK National Health Service (NHS) and Mental Health Foundation and the Mental Health Foundation of New Zealand, for example. With such rich resources, it might be expected that consumer mental-health information needs are being taken care of. But research shows otherwise. This article adds to that research, asking what can be learned about mental-health information needs by directly soliciting questions on mental health from consumers and assessing the extent to which those questions can be answered using a trusted and vetted source of online information. Included in our study is an analysis of the questions in terms of their surface form or structure and the categories they fall into.

It has long been known that the Internet plays an important role in mental-health information seeking, with the lived experience of mental illness being of particular interest (Powell & Clarke, 2006a; Powell & Clarke, 2006b). Khazaal et al. (2008) reported patients searching online for information on specific disorders and substance abuse as well as diagnosis, treatments, and medication side effects. In Lam-Po-Tang& McKay (2010), patients most commonly looked for information on symptoms, treatment, side effects and diagnoses. More recently, a study by Kalckreuth, Trefflich & Rummel-Kluge (2014) confirmed the importance of the Internet to psychiatric patients for information on specific disorders, medication, and mental-health services, and for engaging with other patients and mental-health professionals. Another recent study of psychiatric patients (Trefflich et al., 2015) found the Internet to be an important source of information on medication. There have also been studies of online mental-health information in relation to the general population (Christensen & Griffiths, 2000; Powell & Clarke, 2006b; Leach et al., 2007). The potential of the Internet has been clearly recognized, along with the problems it introduces of information overload and uncertain information quality. As with other Internet-related activities, younger people have been shown more likely to turn to the Internet for mental-health information.

Despite the rich resources of the Internet, and other sources of patient information such as mental-health professionals, the mental-health information needs of patients and the larger community are not being met. In the Kalckreuth, Trefflich & Rummel-Kluge (2014) study, there was no consensus among the patients surveyed that the Internet was of help in managing their disorders. The Khazaal et al. (2008) study concluded that more than half the time the information sought was not found or only partially found, the information that was located was found with relative difficulty, and not all of the information found was comprehensible. There have also been many studies assessing the quality of mental-health Internet sites, from Griffiths & Christensen (2002) to Athanasopoulou et al. (2013) and Monteith, Glenn & Bauer (2013), for example, all reporting on limitations in content, reliability and accessibility.

Unmet information needs in mental health have been identified in two recent studies focused specifically on depression. In a large-scale survey, twenty one specific topics—organized under general information, lived experience, research and policies, and specific treatments—were confirmed as areas of need in the general population (Griffiths & Crisp, 2012). In posts to an online depression support forum, six broad areas of information need were found, namely understanding depression, disclosure and stigma, medication, treatment and services, coping with depression, and comorbid health problems (Barney, Griffiths & Banfield, 2011).

As an alternative to surveys that assess consumers’ knowledge about specific topics in mental health and as an alternative to analyses of online activity such as in mental-health forums, this study proposes that consumers be prompted to ask questions about mental health and that those questions be studied to better understand unmet information needs. No one has investigated what people want to know about mental health by asking them directly. The hypothesis behind this study is that a question-based analysis can provide important insight into what consumers do not know and, by trying to answer those questions using an existing trusted and vetted source of online information, provide an understanding of the extent to which the information needs represented by these questions can or cannot be met online. Directly solicited questions can also be categorized in terms of content and form to provide insight into the information needs they represent.

Clinical information needs have been extensively studied by examining real clinical questions collected from physicians (Ely et al., 2000a; Ely et al., 2000b; Jerome et al., 2001; Huang, Lin & Demner-Fushman, 2006). The questions have further been analyzed to produce taxonomies of question types. These studies have shown that a large percentage of clinical questions correspond to a small number of question types. This understanding has informed the design of clinical information and question–answering systems. No such understanding yet exists for consumer health questions in general or mental-health questions in particular, and no collection of consumer questions on mental health has yet been compiled.

A large-scale source of general consumer health questions is the US National Library of Medicine (NLM). Its customer services receive about 90,000 requests a year from around the world. To provide help in responding to these requests, it recently launched the Consumer Health Information and Question Answering project (Van Der Volgen, Harris & Demner-Fushman, 2013). The project seeks to automatically classify questions and eventually automatically answer them via a question–answer system. Using a sample set of over 11,000 reference questions, 14 categories of health-related consumer questions were proposed including cause, diagnosis, and prognosis, among others. However, the NLM question collection is very broad, with no separate collection of questions on mental-health.

But why questions? Why not analyze the vast amount of data available from search engine queries to understand the mental-health information needs of consumers? Such data can provide and indeed have provided insights, but questions deliver more. Consider the question: *Why do people who use drugs experience more mental illnesses?* (We understand drugs here to refer to illegal or recreational drugs.) The general assumption behind this question is that drug use and mental illness commonly occur together, a fact research shows to be the case (Santucci, 2012). But the form of the question suggests something more, namely that there is or may be a causal link between drug use and mental illness, or that using drugs in some way brings on mental illness. Had the question been *Why do people who experience mental illness use more drugs?* there would have been the suggestion of mental illness possibly leading to drug use, perhaps in an attempt to self-medicate, for example. Such presuppositions are an integral part of the information need even if the questioner is not explicitly aware of them. From the search query *drugs and mental illness* or *mental illness and drugs*, there is no way to know whether either of the two questions was intended or some other, such as *What research has been done on drugs and mental illness?* It is only with a fully expressed question that we understand the real information need.

In a series of NIH-funded projects in the 1990s, research was conducted on new ways to organize and give access to cancer information for both clinicians and consumers (Crangle et al., 1996a; Crangle et al., 1996b; Crangle et al., 1998a; Crangle et al., 1998b; Tuttle et al., 1996). An interesting discovery was that systems designed for clinicians had to be adapted because consumer questions were different from clinical questions in important ways. They were often oriented toward personal experience, for example, and they crossed the topic boundaries found in clinical information.

It is crucial that mental-health information provided to consumers not only include what health professionals know consumers need to know but also take into account what consumers want to know and do not know. Directly solicited questions can reveal this. In addition, directly solicited questions can reveal through their presuppositions what consumers mistakenly believe to be true or are indirectly probing the truth of. In the first and still most recent national assessment of health literacy in the United States, adults at all levels received information about health issues from the Internet (Kutner et al., 2006). Similar figures hold for many other countries; in the UK, for instance, 43% of all adults had used the Internet to find health information online in 2013, and nearly 60% among those aged 25–34 (Office for National Statistics, 2013). A questions-based view of information needs can offer a new perspective on what might need to be included in trusted sources of online mental-health information for consumers.

## Aims

This study set out to collect questions directly from consumers and to analyze them by answering the following three questions: (1) What kinds of mental-health questions do people have? (2) What form do the questions take? and (3) How easily are answers to those questions found in trusted and vetted online sources of information? The aim is to develop new insight into what consumers do not know, how they express their information needs in language, and the extent to which those expressed needs can be met using trusted online information.

## Method

To obtain consumer questions on mental health and illness, we used crowdsourcing; in particular, the platform provided by Amazon Inc.’s Mechanical Turk (AMT). AMT is a digital marketplace that allows workers to perform tasks online in exchange for specified fees. Workers are anonymous to the person requesting the task, as is the requester to the workers. A worker’s unique identifier allows the requester to control the number of times any worker responds to the task request. Results are provided in an Excel spreadsheet that can be downloaded for analysis. AMT has been shown to be a reliable source of participants for clinical studies in consumer health informatics (Carter et al., 2014; Yu et al., 2013). Studies have investigated the quality of data it provides and examined its advantages and disadvantages, concluding that the data obtained are at least as reliable as those obtained via traditional methods (Goodman, Cryder & Cheema, 2013; Paolacci, Chandler & Ipeirotis, 2010; Mason & Suri, 2012). Our questions were solicited through the AMT platform by specifying the following task:

*What do you want to know about depression, anxiety, schizophrenia, bi-polar disorder, suicide or ANY mental-health topic? Enter a full question, not just a phrase. For example: “Do people ever recover from schizophrenia?” NOT ”Schizophrenia recovery.” We do NOT want simple questions of the form “What is X?” where X is a commonly known mental illness. Your question does not have to have perfect grammar but it must be perfectly clear. For example: “Do viruses cause mental illness?” is acceptable. “Is there cause for mental illness?” is not. Spelling mistakes are alright as long as the question is still clear.*

One hundred questions were collected from 100 unique workers between April 26, 2014 5:47 PM PDT and April 26, 2014 9:46 PM PDT for a total cost of $40 and an effective hourly rate for workers of $16. Each worker was allowed to ask only one question to ensure our data represented the information needs of more than those few people who might respond to the AMT task first and provide many questions each, thereby excluding others from offering their questions.

The questions were analyzed three different ways:

1. **Consumer health category analysis:** Using categories adopted from the NLM consumer health question answering project, each question was labelled as to the kind of question it was.
2. **Analysis of question form:** The structure or surface form of each question was investigated.
3. **Analysis of answer accessibility:** An assessment was made as to whether or not each question had an answer that was accessible through the search box at the NIMH site http://www.nimh.nih.gov/.

The questions were analyzed as asked, that is, without correction or editing. The number was limited to 100 to permit a thorough manual search of the trusted and vetted information resources of the NIMH. It is especially time-consuming to establish a negative, namely that a given question cannot be answered from a given document. We discuss at the end of the paper the kinds of automated text analysis that will make further studies of this kind possible with a larger collection of questions. The full set of 100 questions is given in Data S1.

### Consumer health category analysis

Twenty question categories were defined, adapted from the 14 categories of the NLM study of consumer health questions. Adaptions were as follows. In the NLM study, the greatest number of questions (23%) fell in the Management category. This category was replaced by the four NLM subcategories of TREATMENT, CURE, PREVENTION, and OTHER MANAGEMENT to better understand the questions being asked. The Information category had the next highest number of questions (18.1%) in the NLM study. For our study on mental health, any question that appeared to be seeking information to understand mental illness in general or a specific disorder fell into this category. And if a question would require as part of an adequate answer some explanation of mental illness in general or of a specific disorder, it was considered an INFORMATION question. In the NLM study a combined category called Susceptibility had the next highest concentration of questions (14.4%). It was replaced by the three NLM subcategories of DISTRIBUTION of a DISEASE in a POPULATION, INHERITANCE PATTERNS, and TRANSMISSION PATTERNS for infectious diseases. Although the transmission category might not seem justified for mental health, consumers could ask questions that fit the category. Finally, one category was added that is important for mental-health, namely PSYCHOSOCIAL, which covers questions about the social-emotional ramifications of a mental-health disorder. Table 1 presents the 20 categories along with example questions.

**Table 1:** Consumer question categories for mental health.

ANATOMY: questions that refer to a particular part of the body, such as the location affected by a disorder. ***Does the brain damage due to accident or illness make bipolar disorder?***
CAUSE: includes both direct and indirect causes, such as factors that might increase the risk of developing a disorder. ***Do bacteria cause some form of bi-polar disorder to occur?***
COMPLICATION: includes the risks faced by consumers with the disorder but does not include the signs/symptoms of a disorder. ***How does depression in children under 10 years old effect them later in life?***
DIAGNOSIS: includes diagnostic tests or methods for determining the difference between possible diagnoses. ***Is Bi-Polar disorder over diagnosed? What is the difference between bi-polar 1 and 2?***
INFORMATION: includes specific disorders or what counts in general as mental illness. ***Are anxiety disorders and PTSD connected?***
TREATMENT: ***Have SSRI anti-depressants proven to be useless for a lot of people?***
CURE: ***Is anxiety something that you can totally eliminate?***
PREVENTION: ***…mental illness …My father …myself. What to do to get my son free from this mental illness?***
OTHER MANAGEMENT. ***Will I have to stop driving if I have schizophrenia?***
MANIFESTATION: questions asking about signs or symptoms of a disorder. ***What are early warning signs of schizophrenia?***
OTHER EFFECT: questions asking about the effects of a disorder, excluding signs/symptoms or predispositions. *No examples*
PERSON or ORGANIZATION: questions asking for a person or organization associated with a disorder, such as medical specialists, hospitals, or consumer and support groups: *No examples*
PROGNOSIS: includes life expectancy, quality of life, or the probability of success of a given treatment. ***Do you ever really cope with dealing with Depression in every day life?***
DISTRIBUTION of a DISEASE in a POPULATION: ***Why is depression more prevalent in women than in men?***
INHERITANCE PATTERNS: ***Is clinical depression a genetic illness that runs from family member to family member?***
TRANSMISSION PATTERNS for infectious diseases. *No examples*
OTHER: questions that do not belong to the above types. This includes non-medical questions about a disorder, such as requests for financial assistance or the history of a disorder. *No examples*
NOT DISORDER: questions that aren’t about mental health. *No examples*
RESEARCH: questions about the latest research, such as medical publications or clinical trials. ***Are new illnesses still being discovered?***
PSYCHOSOCIAL: questions about the social-emotional ramifications of a mental-health disorder. ***Do people with bipolar disorder or schizophrenia, enjoy more fulfilling romantic relationships with counterparts who also have these mental illnesses?***

A question could receive more than one category label. For example, the question *Is Bi-Polar disorder curable at all or will people always be dependent on their medication?* falls into four categories; namely, CURE, PROGNOSIS, TREATMENT and INFORMATION, the last because the question suggests the need for general information on bipolar disorder.

All 100 questions were categorized by the first author (CEC) and a randomly selected subset of 20 questions was additionally categorized by the second author (JBK). Those 20 questions are indicated by starred rows in Data S1. Inter-rater agreement for this subset was calculated using a chance-corrected measure of agreement modeled on Cohen’s kappa (Cohen, 1960).

### 2. Analysis of question form

It is a fundamental tenet of semantics that meaning does not exist solely within words and sentences but resides in the situations in which the words are uttered and the attitudes of those speaking and hearing the words (Perry & Barwise, 1983). To understand the meaning of a question, three aspects must be considered: the act of asking the question, the words and phrases used to ask the question, and the question itself or its content. The child of a parent diagnosed with schizophrenia may ask: *Is schizophrenia genetic?* The mother of an adult child similarly diagnosed may say: *My father had schizophrenia. Is that why my child has it?* Different acts and different words used to express the question but, in important ways, the same question with the same content, namely an inquiry into the heritability of schizophrenia. However, the first question act may have behind it a concern over whether or not the questioner is at risk. The second may have behind it a concern that she or her family heritage are to “blame” for her child’s illness. The reason for or motivation behind a question is an important part of understanding what is being asked and determining the most appropriate answer.

This study pays attention largely to the second aspect of semantics; namely, the words and phrases used to express the question. This concern with the surface form or structure is not because it is most important but it is where any analysis must start. Most taxonomies of question types, such as those of Lehnert (1977), Lehnert (1978) and Graesser, Person & Huber (1992), assume that questions take simple surface forms such as *Is X true?* (*Are anti-depressants dangerous?*), or *What causes X to occur?* (*What causes PTSD?*). This simplifying assumption may have been necessary for early work on question taxonomy development, but questions real people pose are seldom that simple. The NLM questions project recognizes this reality in its work on decomposing complex questions into their simpler parts (Roberts et al., 2014).

Three complex question forms were identified in this study. A **multi-part question** can be separated into one or more distinct questions. An example is: *What type of life stressors can cause someone to experience depression and what are the first signs of depression?* Two distinct questions can clearly be derived. A **multifaceted question** has a more complex structure. It may present background information necessary to make the question clear. For example, *The brain is divided into multiple parts and the parts are responsible for certain functions that take place in the human body. Which part of the brain is associated with mental illness, depression etc.?* Or it may pose alternatives that are not separable in any simple way. For example, *Is depression caused by something that happens to a person in their past, or is it something the person is born with?* The following two separate questions can certainly be derived: A. *Is depression caused by something that happens to a person in their past?* and B. *Is [depression] something [a] person is born with?* But so can these questions: *Is A true and B false? Is A false and B true? Can both A and B be true?*

A **narrative question** takes the form of a story or a vignette that can give insight into the reason for asking the question. An example is the following: *[Is] mental illness hereditary? My father was a timid and nervous person and so am I. What to do to get my son free from this mental illness?* This question is also multi-part, asking about the inheritability of mental illness, presenting a timid and nervous disposition as connected to mental illness, and asking if the consequences of such a hereditary trait can be bypassed. Questions of complex form can multi-part, multifaceted, and narrative at the same time.

The first author determined the form of the questions as it is within her area of special expertise.

### 3. Analysis of answer accessibility

It is important not only to know what questions consumers have but also whether or not they can find answers to those questions at comprehensive trusted and vetted sources of online information for consumers. The NIMH site (http://www.nimh.nih.gov) was chosen to conduct a review of the extent to which answers are accessible to consumers. Questions were entered into the search box at the NIMH site as they were asked, without any editing or rephrasing. To make the assessment as reasonable as possible, long questions were omitted, thereby precluding many questions of complex form which are naturally more difficult to find answers for. There were 54 short questions (fewer than 255 characters) and these were taken as the test questions. The NIMH website was accessed by CEC on October 17, 2014 and the top three URLs returned for each search were retrieved. A URL (Uniform Resource Locator) is an Internet address that points to a document and it was these documents that were examined for answers to the given questions.

We restricted ourselves to the top three search results because research consistently shows that the first few results returned by a search receive the majority of “clicks,” that is, the link actually gets opened. A recent study found that 53% went to the top result, 15% to the second, and 9 to the third (Goodwin, 2011). Another found that the top result received 36.4%, the second 12.5%, and the third 9.5% (Miller, 2012).

Two assessments were made of answer accessibility. In the first assessment, the first author (CEC) examined the documents that the top three URLs pointed to and made one of the following three judgments for each question:

An answer could not be found in the top three documents returned, or no results were returned.

An answer could be found but it was in a long or complex document that required extensive reading and some interpretation.

An answer could be found quite easily in the documents returned, starting with the first, moving on to the second or third as needed.

In the second assessment, AMT workers were used. For each question an AMT task was set in which the question was presented along with the top URL retrieved by CEC earlier. The worker was asked to indicate if an answer could be found in the document the URL pointed to by following the link and copying and pasting an excerpt containing the answer in a text box or, if no answer could be found, to enter NO ANSWER, or if no document was returned to enter NO DOC. Each task was performed by two workers on February 5, 2015, for a total cost of $76.20. Workers spent longer than expected on each task—on average 13 minutes compared to the 4 minutes expected—giving a lower than intended effective hourly rate of $3.79.

Given this average time of 13 minutes, we limited the AMT worker assessment to just the top search result. The length of time it took to ascertain whether or not there was an answer in a document is itself a result that establishes the difficulty of the task for the consumer. We discuss this further in the Results section.

## Results

### 1. Consumer health category analysis

For the categorization of all 100 questions by CEC, Table 2 shows the results. Each question could, and many did, receive more than one label. The most common label was INFORMATION and it applied to 81 questions. Two label clusters occurred. There were 24, 23, 22, and 21 questions respectively labelled TREATMENT, CAUSE, MANIFESTATION, and PROGNOSIS. And there were 13, 13, 12, and 12 questions respectively labeled COMPLICATION, INHERITANCE PATTERNS, CURE, and PSYCHOSOCIAL. A total of 248 labels were applied to the 100 questions, which gave an average label density of 2 per question. Thirteen questions had only one category label.

**Table 2:** Numbers of questions receiving each category label.

Category	Number of questions (out of 100) receiving this category label
INFORMATION	81
TREATMENT	24
CAUSE	23
MANIFESTATION	22
PROGNOSIS	21
COMPLICATION	13
INHERITANCE PATTERNS	13
CURE	12
PSYCHOSOCIAL	12
DIAGNOSIS	9
DISTRIBUTION of DISEASE	8
ANATOMY	4
OTHER MANAGEMENT	4
PREVENTION	1
RESEARCH	1
OTHER EFFECT	0
PERSON or ORGANIZATION	0
TRANSMISSION PATTERNS	0
OTHER	0
NOT DISORDER	0

For the 20 randomly selected questions that were additionally categorized by JBK, we computed inter-rater agreement using the chance-adjusted measure according to the formula *k* = (Na − Nc)/(Np − Nc), where Na = number of agreements overall, Nc = number of agreements that could be expected due to chance, and Np = maximum number of agreements possible. An agreement for a question is an instance in which a given category is either assigned by both raters to that question or by neither. A disagreement for a question is an instance in which a given category is assigned by one rater to the question but not by the other. The number of agreements overall is the sum of the agreements for all 20 questions. With 20 questions and 20 categories, the maximum number of agreements possible is 20 × 20. Chance assignment of a category to a given question is 0.5 for each of the 20 categories, which sums to 0.5 × 20 = 10 for each question. For 20 questions, the number of agreements due to chance is therefore 10 × 20 = 200.

The agreements and disagreements are shown in Table 3. The question numbers given at the top of the columns correspond to the numbers in the list of all 100 questions given in Data S1. Each element of the matrix indicates whether neither (blank), one (1), or both (2) raters assigned the category given by the row to the question given by the column. A blank (both did not assign that category) or a 2 (both did assign that category) indicates agreement, a 1 (only one of the raters assigned that category) disagreement.

**Table 3:** Matrix of agreements and disagreements by two raters for the assignment of categories to 20 questions.

	Question																			
Category	*5*	*17*	*20*	*22*	*25*	*28*	*34*	*38*	*40*	*44*	*45*	*50*	*55*	*63*	*68*	*81*	*83*	*89*	*91*	*93*
ANATOMY			2													2				
CAUSE	2		1	2			2													
COMPLICATION			1																2	
DIAGNOSIS		2	1								2									
INFORMATION	1	2	2	2	2	2	2	2	2	2	1	2	2	2	2	2	2	2	2	2
TREATMENT					2	2	1	2		2			2					1		2
CURE							2	1					2							2
PREVENTION				1																
OTHER MANAGEMENT																		1		
MANIFESTATION		2							2		2	2		2	2					
OTHER EFFECT		1							1					1						
PERSON / ORG																				
PROGNOSIS					2			2	1				2		2				1	
DISTRIBUTION …																				
INHERITANCE …	1																2			
TRANSMISSION …																				
OTHER																				
NOT DISORDER																				
RESEARCH																				
PSYCHOSOCIAL																				
ANATOMY		1	1															1		

Taking all 20 questions and categories, we obtained *k* = 0.9. If we omit the INFORMATION category, which had a disproportionally high number of agreements, the score stays the same. If we omit the categories shown by shaded row labels in the Table that were not relevant to the subset of 20 questions and so also contributed a high number of agreements, we again get a score of 0.9.

### 2. Analysis of question form

The majority of the questions (74 out of 100) are simple in form. Twenty six are complex, with 11 being multi-part, 9 multifaceted and 6 narrative, as shown in Table 4. (The 26 questions are numbered consecutively and not according to their place in Data S1). One question, the last in the Table, is both multi-part and narrative but listed only under the Narrative category. The multi-part questions can each be parsed into two or more separable simple questions. The multi-faceted questions can often also be split into separate questions but they present additional challenges in their interpretation. Even a question as apparently simple as 14—*Is a predisposition for bi-polar disorder hereditary or can it be caused by environmental factors?*—may have an exclusive-or or an inclusive-or interpretation and so is not merely two simple questions. That is, the question may have any of the following three summarized interpretations: *Is there an inherited predisposition and no environmental effect? Are environmental factors the cause and a disposition is not inherited? Do inherited and environmental factors both play a causal role?*

**Table 4:** Questions of complex form.

**Multi-part questions**
(1) *What are the symptoms of schizophrenia & is there immediate cure for it in the starting stages?*
(2) *What are the long term effects of the over-diagnosis of ADD in both adults and children?*
(3) *I have wondered if Anxiety is a case of having fear for some things like feeling attack by loudness of a person or Nervousness of a interview. Can Anxiety be a normal thing for adults 21 and above?*
(4) *Can someone have more than one mental illness at the same time? Are new illnesses still being discovered?*
(5) *How is mental illness generally caused and how long it would prevail and what are the ways to treat it?*
(6) *Is it safe to marry an individual who is or was treated with mental disorders? What is the chance in percentage that the children out of this relation will have the same disease?*
(7) *What causes depression, if anything? Does medicine really help against depression?*
(8) *What type of life stressors can cause someone to experience depression? And what are the first signs of depression?*
(9) *Does the mental patients have a tendency to commit suicide and why? It is said that people who had multiple surgeries suffer mental illness. Is it true?*
(10) *What is the best possible way that a person can recover from anxiety disorder, and is medication the best way to achieve such success?*
(11) *Do people ever recover from what I believe may be a rare mental ailment called Erotomania, and what may be the cause of this condition?*
**Multifaceted questions**
(12) *How will a person overcome social anxiety without medications over time or in a short period if possible?*
(13) *How can a mental disorder be considered psychological if they find a neurological basis for the disorder? Even if it has effects on the personality, doesn’t that still make it a neurological problem?*
(14) *Is a predisposition for bi-polar disorder hereditary or can it be caused by environmental factors?*
(15) *Is depression is a cause of mental illness? How can one get rid of such illness it at all it is an illness?*
(16) *Do people who suffer from schizophrenia hear voices of people they know (or themselves) or are they voices that they don’t recognize?*
(17) *Do people with bipolar disorder or schizophrenia, enjoy more fulfilling romantic relationships with counterparts who also have these mental illnesses?*
(18) *Can people with down syndrome go to colleges or universities and succeed despite of their low IQ? Can they succeed at hard courses (like Calculus, Organic Chemistry, Microbiology…)?*
(19) *Is anxiety attributed more to genetics and “wiring” of the human brain, or conditions presented during developmental years?*
(20) *Is depression caused by something that happens to a person in their past, or is it something the person is born with?*
**Narrative questions**
(21) *I would love to know is it possible for someone with bi polar who has been unfaithful as part of a maniac episode will they ever learn to be faithful again? Or is that always going to be a symptom of their maniac episodes.*
(22) *As a child my mother displayed some attributes that I believed to be mental illness. These characteristics included, laughing at me when I had a bike wreck that busted my lip, getting me up late at night to play the piano for her to sing because she could not play (school nights), calling my sister names i.e., lazy or sorry and she would spell the names and sing it at my sister while hitting her, she laughs at life events that are sad and does not cry when close family members pass, but adores my only younger brother??? My sister and I have very deep scars from her actions but she was never treated for mental illness. Is this behavior she displays mental illness? She has not changed today she is older.*
(23) *In ones life when living for Christ, many believe that these kinds of conditions are brought on by demonic influences. Does anyone ever think to call upon the Lord God for deliverance in these cases?*
(24) *Whenever i feel very disappointed because of failure, or humiliated, i feel like i should suicide and i start thinking of which is the easiest way to do so. Is it normal?*
(25) *if someone who went through a 2 week episode of major depressive disorder then is he/she likely to expereince that 2 week of depressive mode again?*
(26) *Are mental illness hereditary? My father was a timid and nervous person so do myself. What to do to get my son free from this mental illness?*

Complex questions present another challenge, not so much for their interpretation but for finding answers. Complex questions often include information that is very specific, such as being unfaithful or disappointed, experiencing failure, experiencing a two-week episode, or having a timid father. Although it is often possible to formulate general answers that take specifics into account, search and retrieval technology behind search boxes at websites cannot make the leap from the specific to the general. Nor can the consumer always make the necessary generalization; to know that timidity, for instance, is not a personality trait that generally shows up in discussions of mental health.

### 3. Analysis of answer accessibility

The first assessment of answer accessibility (by CEC) had the results shown in Table 5 (where the 54 questions are numbered consecutively and not according to their place in Data S1). For 22 of the 54 questions an answer could be found in the top documents returned. For 14 of the 22, the answer was easy to find. For the other eight, the answer was in a long or complex publication that required some interpretation. For the remaining 32 questions, no answer could be found, and for seven of those questions no search results at all were returned.

**Table 5:** Questions and their answer status.

**Questions with no answers and questionable presuppositions**
(1) *Why do people who use drugs experience more mental illnesses?*
Assumption: Drug use (illegal or recreational) and mental illness commonly co-occur, an assumption supported by research. However, more specifically, from the form of the question, there is the assumption that people who use drugs experience more mental illness than those who don’t use drugs and there may be a causal link between drugs use and mental illness.
(2) *Have SSRI anti-depressants proven to be useless for a lot of people?*
Assumption: SSRIs have proven useless for some people.
(3) *What age group is most susceptible to anxiety in the United States?*
Assumption: There is an age range during which a person is most susceptible to anxiety in the United States.
(4) *Do patients every completely recover from narcissistic personality disorder?*
Assumption: Complete recovery is something that can be predicted in mental illness.
(5) *How does depression harm the brain?*
Assumption: Depression causes known harm to the brain.
(6) *Is recovery from depression based on a decision to be more positive?*
Assumption: Being positive can bring about recovery from depression.
(7) *What is the best natural remedy for Anxiety?*
Assumption: There are natural remedies for anxiety and one can be identified as the best.
(8) *Can mental illnesses be cured by detoxing heavy metals?*
Assumption: Heavy metal detoxification has been the subject of mental-health research.
(9) *Suicide is most common with which mental illnesses?*
Assumption: Enough is known about suicide to associate its occurrence with specific mental illnesses.
(10) *Is depression linked to a certain gene since it does seem to run in families?*
Assumption: One specific gene may be responsible for depression.
(11) *How does depression in children under 10 years old effect them later in life?*
Assumption: Depression in childhood has known effects that last into adulthood.
**Additional questions with no answer**
(12) *Can depression be treated with non-pharmaceutical drugs?*
(13) *Is bi-polar disorder more common in young males than in young females?*
(14) *Can severe depression and anxiety ever be completely managed without medication?*
(15) *Do bacteria cause some form of bipolar disorder to occur?*
(16) *Can Schizophrenia be hereditarily transmitted?*
(17) *Is anxiety something that you can totally eliminate?*
(18) *Are there any alternative treatment options for schizophrenia?*
(19) *What is the difference between Neurosis and Psychosis?*
(20) *Can borderline personality disorder be passed down genetically?*
(21) *Can mental illness be caused by a poor diet?*
(22) *Is Alzheimer’s disease hereditary?*
(23) *What is the difference between bi-polar 1 and 2?*
(24) *How long does it typically take for Post Partum Depression to resolve itself?*
(25) *Can you recover from mental illness without the help of traditional medicine?*
(26) *Do thoughts of suicide go away by them selves or do you need a doctors help?*
(27) *Does depression ever fully go away?*
(28) *Is depression manageable solely through talk therapy (with a psychiatrist)?*
(29) *Do you ever really cope with dealing with Depression in every day life?*
(30) *What internal coping mechanisms can you use to help deal with bi-polar disorder?*
(31) *Is post traumatic stress disorder permanent?*
(32) *Does the brain damage due to accident or illness make bipolar disorder?*
**Questions with answers that were there but difficult to find**
(33) *Are people genetically predisposed to specific mental illnesses?*
(34) *Will I have to stop driving if I have schizophrenia?*
(35) *Is depression hereditary?*
(36) *Is anxiety a form of depression? What triggers anxiety?*
(37) *Can depression be associated with pregnancy?*
(38) *What are some ways to conquer anxiety?*
(39) *How early do individuals with schizophrenia begin to manifest symptoms?*
(40) *Are anxiety disorders and PTSD connected?*
**Questions with easily found answers**
(41) *What causes mental illness to occur in people?*
(42) *Can depression weaken a person’s immune system?*
(43) *Why is depression more prevalent in women than in men?*
(44) *What is the relationship between circadian rhythms and Bipolar Disorder?*
(45) *What are early warning signs of schizophrenia?*
(46) *What is the best way to treat bi-polar disorder?*
(47) *Is Bi-Polar disorder over diagnosed?*
(48) *What are the symptoms of the bi-polar disorder?*
(49) *What are the most common symptoms of anxiety?*
(50) *At what age do symptoms of schizophrenia usually begin to show in those affected?*
(51) *How do you know if your loved one has depression?*
(52) *How do you treat OCD?*
(53) *Does schizophrenia only happen to people over a certain age?*
(54) *Are people with schizophrenia violent?*

Twenty of the fifty five top URLs that were returned pointed to consumer-oriented material organized under Health Topics at the NIMH website—such as the following, for example: http://www.nimh.nih.gov/health/topics/depression/index.shtml (accessed 6 February 2015). Some but not all yielded answers to the specific question posed. Sixteen URLs pointed to news items on the NIMH site, such as the following: http://www.nimh.nih.gov/news/science-news/science-news-about-depression.shtml (accessed 6 February 2015). Although an answer might lie within such a document, it was typically difficult to find among all the news article links given, which had to be followed individually. Some URLs pointed to documents such as the following 2011 Director’s Report, which is not consumer-oriented and did not contain an answer: http://www.nimh.nih.gov/about/advisory-boards-and-groups/namhc/2011/september/directors-report.shtml (accessed 6 February 2015). Some URLs pointed to a long pdf (portable document format) document, such as the following budget report, which did not provide an answer: http://www.nimh.nih.gov/about/budget/cj2007_33873.pdf (accessed 6 February 2015).

A number of the questions entailed presuppositions that are not necessarily true and which may have precluded an answer being found at any trusted and vetted information site. Take, for example, the question *On average, how much time does a person suffering from bi-polar disorder spend in a manic state compared to a depressive state?* The underlying assumption is that it is possible to predict the relative lengths of manic and depressive states in general. The question *Suicide is most common with which mental illnesses?* assumes enough is known about suicide to associate its occurrence with specific mental illnesses in a quantifiable way. Such presuppositions can be identified for 11 of the 32 questions without an answer and these are given at the top of Table 5. It is not immediately obvious from a question’s presuppositions whether it will or will not have an answer. It depends on what research has been done. For example, the question *What is the relationship between circadian rhythms and Bipolar Disorder?* assumes there is a relation between circadian rhythms and mental illness and that it has been studied. In fact, the NIMH search returns a report on the topic from an NIMH workshop that does provide an answer to the question.

The second assessment of answer accessibility, conducted by AMT workers, had similar results, with answers found by both or one of the AMT workers for 20 of the questions. However, the AMT workers failed to find answers to questions numbered 36, 38, 39 and 53 in Table 5 but did produce excerpts for the questions numbered 7 and 11. These excerpts arguably provide answers that some consumers might find sufficient.

Question 7: Stress management techniques and meditation can help people with anxiety disorders …, aerobic exercise may have a calming effect. …, certain illicit drugs, and even some over-the-counter cold medications can aggravate ….

Question 11: Children who develop depression often continue to have episodes as they enter adulthood. Children who have depression also are more likely to have other more severe illnesses in adulthood.

In two instances of answers not being found, the URL pointed to a pdf file which produced the following standard warning before it was opened:

Opening <filename>.pdf. Some files can contain viruses or be otherwise harmful to your computer. It is important to be certain that this file is from a trustworthy source. Would you like to open this file?

The AMT workers most likely declined to open such a file, which accounts for their reporting the result of NO DOC (no document returned).

Inter-rater agreement of *k* = 0.8 between the first and second assessment was calculated using Cohen’s kappa, computed as *k* = (Pa − Pe)/(1 − Pe) on the results in Table 6, where Pa is the probability of both assessments classifying a randomly selected question the same (that is, both having an ANSWER or both having NO ANSWER) and Pe is the probability of both assessments classifying a randomly selected question the same by chance.

**Table 6:** Matrix of inter-rater agreement for first and second assessment of answers.

	First assessment	
Second assessment	ANSWER	NO ANSWER
ANSWER	18	2
NO ANSWER	4	30

The length of time it took for the AMT workers on average to ascertain whether or not there was an answer in a document—13 minutes—made us decide to restrict the second assessment to just the top document. If it takes that long to check one document it seems unlikely that consumers would be prepared to search one document after another for an answer. Despite the difference in the number of documents used in the first and second assessments, there was still high inter-rater agreement. This suggests that much of the time if an answer exists, it can be found in the first, second and/or third documents, just in different forms.

## Conclusions

### 1. Consumer health category analysis

The high percentage of mental-health questions categorized under INFORMATION—81% compared to 18.1% for the general consumer health questions from the NLM—suggests that consumer grasp of mental illness is low compared to other health topics. Questions sought both general information and information on specific disorders, notably depression (29 questions), bipolar disorder (16), anxiety (14) and schizophrenia (12). These four disorders were named in the AMT task description, which may have increased the likelihood of their being specifically asked about. However, in the US anxiety and depression have the highest 12-month prevalence figures for adults and the four named disorders are among the top six contributors to disability-adjusted life years for mental and behavioral disorders, with drug and alcohol use disorders being the other two (NIMH, Health and Education, Statistics, http://www.nimh.nih.gov/health/statistics/index.shtml, accessed 20 January 2015). Nonetheless, any follow-up study collecting questions should mention all disorders that are of interest to the study or none, and should explicitly include or exclude substance abuse disorders. Other disorders specifically named in the collected questions were ADD, DID (Dissociative Identity Disorder formally multiple personality disorder), narcissistic personality disorder, “Neurosis and Psychosis,” borderline personality disorder, drug use, OCD, Erotomania, and PTSD (twice).

In many of the studies cited in the Background Section, medication and its side effects were singled out as a topic of specific interest in mental health. The NLM consumer question categories do not separate medication from other treatments, but if we examine our questions we find 12 or 13 questions about medication, depending on how you interpret the phrase *traditional medicine*. This puts the topic of medication in the second cluster of categories. The first cluster, with the greatest frequencies apart from INFORMATION, contained TREATMENT, CAUSE, MANIFESTATION, and PROGNOSIS. The second cluster contained COMPLICATION, INHERITANCE PATTERNS, CURE, and PSYCHOSOCIAL.

Earlier studies also identified a need for information on lived experience, symptoms, diagnosis, and treatments. And in the study of posts to an online support forum, additional topics were understanding depression, disclosure and stigma, coping with depression, and comorbid health problems. The questions labeled PSYCHOSOCIAL (second cluster) in our study were questions about the lived experience of mental illness for the patient and other affected individuals. The category MANIFESTATION (first cluster) is largely about symptoms and these questions were common, as were questions labeled TREATMENT (first cluster). There were some but not many (nine) questions labeled DIAGNOSIS, which was not in the top two category clusters. There were many questions labeled CAUSE (first cluster). The prevalence of these questions suggests a strong need to understand the origins of mental illness. The CAUSE questions overlap with those categorized under INHERITANCE PATTERNS (second cluster). Taken together the questions in these two categories were twice as frequent as those in any other category besides INFORMATION. There were seven questions about stigma, four asking if something is “normal,” one specifically mentioning stigma, one asking about violence, and one questioning the safety of marriage to a person with a mental illness. There weren’t any questions on disclosure in our study. The many questions combined under PROGNOSIS (first cluster), COMPLICATION (second cluster), and CURE (second cluster) would be aligned with the topics of coping and comorbid health problems identified in earlier studies. Despite the fact that the topics of earlier studies and the NLM categories are not fully aligned, there seems to be broad agreement on the content of consumer questions, but with a lesser emphasis on diagnosis and disclosure in our question collection.

There was strong agreement between the two raters on the categorization of the questions. Differences that did exist revealed something about the process of categorization. Interpreting a question to determine which categories applied to it entailed making assumptions about what the questioner may have had in mind and what an adequate answer to the question would entail. For example, for the question numbered 89 in Data S1, *What internal coping mechanisms can you use to help deal with bi-polar disorder?*, CEC assumed that by “internal coping mechanisms” the questioner meant things she could do herself without help from health-care professionals and so categorized it as a PSYCHOSOCIAL question whereas JBK linked “internal coping mechanisms” with cognitive behavioral therapy and so categorized the question as a TREATMENT question. For question 22, *How does poor nutrition during childhood affect one’s susceptibility to develop bipolar or some other mood disorder like depression?*, JKB assumed the questioner had in mind that good nutrition might confer a preventive advantage and so assigned the category PREVENTION. For question 5, *What causes mental illness to occur in people?* JKB determined that an adequate answer would entail some discussion of genetic factors and so assigned the category INHERITANCE PATTERNS.

### 2. Analysis of question form

In this initial study, question form analysis was restricted to the top-level characterization of simple, multi-part, multifaceted or narrative. Despite the fact that the majority of questions were simple in form, categorization was not simple and finding answers was not simple. Questions that are simple in form can still have complex meaning. An example is question number 1 in Data S1: *Why is there a stigma if you have Bipolar Disorder that all of those who have it must be crazy?* A grammatically correct version of this question would be *Why do people hold the stigmatizing belief that a person with bipolar disorder is “crazy”?* This is in the first place a question about stigma, about trying to understand the origins of or reasons behind the stigma of mental illness. But it is also about particular attitudes towards a particular disorder, namely that the person with bipolar disorder is a different, stranger, lesser person, as suggested by the colloquial term *crazy*.

The form and content of questions have long been studied from the perspective of education. The aim has been to understand what questions to ask students that will best guide their learning. A further aim has been to glean from student questions what they need to learn. An early and oft-cited work is that by Bloom et al. (1956) with a more recent example given by Dantonio & Beisenherz (2001). It is well understood from these studies that the form of a question reveals something about the current knowledge of the questioner. A question such as *Are X and Y related?* implies a different level of knowledge from *How are X and Y related?* The first question shows uncertainty as to whether a relation exists while the second presumes a relation and seeks to know more about it. The form of the question therefore constrains the kind of answer that is appropriate and relevant to the information need. For these reasons, further study of consumer questions on mental health needs to include a deeper analysis of the question form, one that can profit from the large body of research in education.

### 3. Analysis of answer accessibility

The NIMH provides extensive patient-oriented material and supports research on almost all the topics addressed by the collected questions (http://www.nimh.nih.gov/health/topics/index.shtml, accessed 20 January 2015). However, answers to more than half of the questions were not readily available. Unanswered questions came from any category and included questions that were both simple and complex in form. Admittedly, answers satisfactory to the AMT workers may have been found in the second or third document returned, which research shows is examined around 24–32% of the time. If answers were to be found in the fourth and later documents not checked by CEC, it is less likely these would be found by consumers. There is the possibility that by adding the online resources of trusted consumer organizations such as NAMI and The Mental Health Foundation of New Zealand, more answers would be found. But at the same time a higher number of irrelevant documents would likely be returned as well, the core problem of the online searching experience. Nevertheless, the restriction to the top documents is an acknowledged limitation of the current study.

What we take away from this analysis is not just that a significant number of answers are not readily available. It is what we can learn from the probable reasons for the failure to find answers. There are four: troublesome presuppositions in the questions; language precision; surface-form search sensitivity; and question specificity. The first and fourth reasons point to the need to collect consumer questions on a larger scale to enable further study. The second and third reasons point to the inadequacies of search technology for matching a question to text in documents.

First, a question may have a presupposition that makes the question unanswerable. For example, the question *What age group is most susceptible to anxiety in the United States?* (numbered 33 in Data S1) presupposes that there is an age range during which a person is most susceptible to anxiety and that research has been done to ascertain what it is in the United States.

The second reason a question may not find an answer is related to language. There is information at the NIMH site about the prevalence of anxiety in different age groups and it may very well be that the questioner would be satisfied with information about prevalence. It was the word *susceptibility*, however, that the search process attempted to match along with the words *age* and *anxiety*. There is the well-known problem of specialist medical terminology that a layperson is not familiar with and layperson terms, such as *nerve medication* (question 94), that are not used in professional medicine. Here however, we have the problem of precision. The distinction between susceptibility and prevalence important to question 33 is not one that can be elided and carefully written material will not confuse the two. But if the act of asking the question is properly understood, that is, if the questioner’s purpose in asking the question is taken into account, the conceptual overlap between susceptibility and prevalence might be acknowledged and a suitable answer given about the prevalence of anxiety in different age groups.

A third reason for answers not being found has to do with the surface form of questions and the sensitivity of the search process to the surface form. Take the question *What is the difference between bi-polar 1 and 2?* Taking the three top documents returned at the NIMH site for this question, two were about an NIMH Strategic Plan and one was a report from a workshop on mental health and mass violence. The phrase *difference between* and the word *bi-polar* were matched to the content in these documents and nowhere, it appears, did the words *bi-polar 1 and 2* appear in a document that used the word *difference*.

To appreciate how important the actual phrasing of the question is to the likelihood of an answer being found through the search box, consider the following two questions, both asking about the onset of schizophrenia symptoms. The first question did have an easily found answer in the top document: *At what age do symptoms of schizophrenia usually begin to show in those affected?* But the second question did not: *How early do individuals with schizophrenia begin to manifest symptoms?* The most likely reason for the difference is that there are direct discussions of age of onset but these do not use the word *early*.

A **f**ourth reason consumer questions may not readily find answers lies in their specificity. “Being more positive,” “children under 10,” “detoxing heavy metals”—these specific references are unlikely to find an exact match in consumer information materials even if there are discussions of patient attitudes and children and toxic metals and illness. The narrative questions all include details that are specific to someone’s circumstances. Such questions will require individualized answers. Is it worth the effort to provide them? That remains to be seen but it is likely that the stories they tell will provide insight for others in similar circumstances. The studies by Powell & Clarke (2006a), Powell & Clarke (2006b), Kalckreuth, Trefflich & Rummel-Kluge (2014) and Griffiths & Crisp (2012) all recognized the value of direct reports from consumers of lived experience.

In looking at the high number of questions for which answers could not be found, it might be argued that consumers ask the “wrong” questions. That view, however, does not respect the right of consumers to have information needs of any nature and to express those needs in whatever way they are able.

## Discussion

This study aimed to contribute to the research on consumer mental-health information by soliciting consumer questions directly, categorizing them, analyzing their form, and assessing the extent to which they can be answered from a trusted and vetted source of online information. The Internet is a much-used resource for health information by consumers. According to a recent Pew report, 59% of U.S. adults, and 72% of Internet users, looked online for health information in the previous year (Fox, 2013). The downside to this wealth of health information online and the easy access to it are clearly known. In evaluating online health information seeking behavior, Fiksdal et al. (2014) discuss the user process of searching, filtering, and comparing results, describing “drawn-out user-driven comparisons of content obtained from multiple sources of varying quality and unverified validity.” Information saturation and fatigue are the result and, with some sources of information unable to be trusted without independent vetting, the consumer can be left confused and unsure. In a comprehensive recent review of interventions to help consumers find and understand reliable and relevant online health information, Lee et al. (2014) report a paucity of research in this area despite the importance of the need.

Given the need for more research, what is the significance of the study reported in this paper? What do we know about mental-health information and consumers that we did not know before?

We have shown that even highly trusted and well-organized sources of information such as the NIMH may not readily provide answers to consumer questions online. Two kinds of explanations presented themselves. First, the clear inadequacies in the search and information retrieval technology deployed at Internet resources such as those of the NIMH. There is no easy fix for this. The problem of matching a question expressed in ordinary language to the text in documents is a long-standing and active area of research on a non-trivial problem, as demonstrated by the 24-year history of the Text Retrieval Conference (TREC) workshop series, which encourages research in information retrieval by providing large test collections for tasks such as the retrieval of documents on the Internet and question answering (http://trec.nist.gov). Second, the nature of consumer questions in mental health mitigates against the easy provision of answers. There are presuppositions that consumer questions harbor; a question that has a false presupposition is less likely to have an answer in trusted and vetted sources of information. Consumer questions are also expressed with a degree of specificity that makes the retrieval of relevant information difficult.

But there is another reason consumer questions do not find ready answers. We do not yet know what those questions are. Until we do large-scale and systematic collection of questions we will not know.

We have shown in this study that it is informative to ask consumers directly what they want to know. Conducting surveys that probe consumers’ grasp of specific topics is informative, analyzing the content of online consumer groups yields essential information, and examining the history of search terms entered at search-engine interfaces gives insight. But questions directly solicited from consumers have their own contribution to make.

With a large enough collection of consumer questions, quantitative analytic methods can be deployed. We end with a brief discussion of what these methods offer. Quantitative text analysis has been used to do text data mining in the scientific literature (Crangle et al., 2007) and detect patterns in language for studies of the brain (Crangle, Perreau-Guimaraes & Suppes, 2013; Crangle, 2014). An alternative to qualitative methods such as those described in Barney, Griffiths & Banfield (2011) and Griffiths & Crisp (2012), quantitative methods applied to language also allow common themes to be identified. Taking our 100-question collection we can find *n*-grams, which are sequences of *n* (typically two or three or four) words that occur together more often than would be expected by chance. The only four-word n-gram of note in our 100 questions is *what is the best* and, apart from the phrases *mental illness(es), bi(-)polar disorder,* and *the brain*, the other noteworthy bi-grams are *the best*, *symptoms of*, and *recover from*. It appears to be a common theme that consumers want answers that provide comparative results, as indicated by *the best*. An interest in diagnosis may be suggested by *symptoms of*, but it may also suggest attempts to understand the experience of someone with a mental illness, wanting to know if observed behavior is a manifestation of a disorder or not. An intuitive reading of the questions as a whole supports the idea that the bi-gram *recover from* gives voice to a hope that mental illness can be recovered from, as opposed to being managed as a chronic condition. These observations are tentative given the relatively small collection of questions but they point to the potential of large-scale question collection for robust quantitative analysis. Question should be collected in a number of different contexts, including online at a consumer organization such as NAMI, through crowdsourcing, and at locations used in other studies—psychiatrists’ offices and hospitals, for example.

With sufficient consumer questions, quantitative methods can also be deployed to identify common presuppositions in questions. Understanding common presuppositions could lead to a better understanding of the knowledge consumers may need but may not realize they need. Understanding common presuppositions also reveals the questions that must be addressed at trusted and vetted online sites, even if just to point out there is no definitive answer. Take the question *How does depression harm the brain*? Because the presupposition—that depression does indeed harm the brain—does not have a consensus evidence-based opinion, an answer will not be found for consumers in trusted and vetted information sources. But using a general-purpose search engine, a consumer will find many “answers” to the question at less trusted and non-vetted online information sources. It is imperative that the most trusted online sources of mental-health information be the ones that consumers go to, because it is at those sources that their questions can be answered.

## Supplemental Information

10.7717/peerj.867/supp-1 Data S1 Patient Questions Supplemental Data
