# Supplementary material for: A questions-based investigation of consumer mental-health information
Source: PeerJ. 2015 Mar 31;3:e867. doi: 10.7717/peerj.867 (PMC4393807; doi:10.7717/peerj.867)
Supplement: Data S1 [file peerj-03-867-s001.pdf]

S1: List of 100 consumer questions on mental health

Starred question numbers indicate the 20 questions used to determine inter-rater reliability for the categorization of questions. The rows with formatting in bold and italics show the questions used in the analysis of answer accessibility.

1	Why is there a stigma if you have Bipolar Disorder that all of those who have it must be crazy?
2	What is the best type of treatment for someone who suffers from schizophrenia? (allows them to live the most success, independent lives)
3	***Why do people who use drugs experience more mental illnesses?***
4	Is clinical depression a genetic illness that runs from family member to family member?
*5**	***what causes mental illness to occur in people?***
6	Is there any types of natural or herbal medicines or remedies to help treat mental illnesses that one may suffer from.
7	WHAT ARE THE SYMPTOMS OF SCHIZOPHRENIA & IS THERE IMMEDIATE CURE FOR IT IN THE STARTING STAGES?
8	What would you recommend a friend or loved one do to help someone suffering from clinical depression?
9	***Can depression weaken a person's immune system?***
10	***Why is depression more prevalent in women than in men?***
11	What are the long term effects of the over-diagnosis of ADD in both adults and children?
12	***What is the relationship between circadian rhythms and Bipolar Disorder?***
13	How will a person overcome social anxiety without medications over time or in a short period if possible?
14	***Are people genetically predisposed to specific mental illnesses?***
15	Can people who have DID (formally MPD, multiple personality disorder) ever really integrate and lead normal lives?
16	I would love to know is it possible for someone with bi polar who has been unfaithful as part of a maniac episode will they ever learn to be faithful again? Or is that always going to be a symptom of their maniac episodes.
17*	As a child my mother displayed some attributes that I believed to be mental illness. These characteristics included, laughing at me when I had a bike wreck that busted my lip, getting me up late at night to play the piano for her to sing because she could not play(school nights),calling my sister names i.e. lazy or sorry and she would spell the names and sing it at my sister while hitting her, she laughs at life events that are sad and does not cry when close family members pass, but adores my only younger brother??? My sister and I have very deep scars from her actions but she was never treated for mental illness. Is this behavior she displays mental illness? She has not changed today she is older.
18	***Can depression be treated with non-pharmaceutical drugs?***
19	I have wondered if Anxiety is a case of having fear for some things like feeling attack by loudness of a person or Nervousness of a interview. Can Anxiety be a normal thing for adults 21 and above?

20*	How can a mental disorder be considered psychological if they find a neurological basis for the disorder? Even if it has effects on the personality, doesn't that still make it a neurological problem?
21	Is a predisposition for bi-polar disorder hereditary or can it be caused by environmental factors?
22*	How does poor nutrition during childhood affect one's susceptibility to develop bipolar or some other mood disorder like depression?
23	***What are early warning signs of schizophrenia?***
24	***Is bi-polar disorder more common in young males than in young females?***
25*	***Can severe depression and anxiety ever be completely managed without medication?***
26	***Will I have to stop driving if I have schizophrenia?***
27	Is mental illness a hereditary disease that is transmitted from one generation to other ?
28*	***What is the best way to treat bi-polar disorder?***
29	What causes persons to compulsively harm themselves physically, such as cutting and burning themselves?
30	***Have SSRI anti-depressants proven to be useless for a lot of people?***
31	***Do bacteria cause some form of bi-polar disorder to occur?***
32	***Is Bi-polar disorder over diagnosed?***
33	***What age group is most susceptible to anxiety in the United States?***
34*	Is depression is a cause of mental illness? How can one get rid of such illness it at all it is an illness ?.
35	Can someone have more than one mental illness at the same time? Are new illnesses still being discovered?
36	***Can Schizophrenia be hereditarily transmitted?***
37	how is mental illness generally caused and how long it would prevail and what are the ways to treat it?
38*	If a person takes antidepressants and sees a therapist for depression will she have to continue doing both for the rest of her life?
39	***Is anxiety something that you can totally eliminate?***
40*	On average, how much time does a person suffering from bi-polar disorder spend in a manic state compared to a depressive state?
41	***Do patients every completely recover from narcissistic personality disorder?***
42	***What are the symptoms of the bi-polar disorder?***
43	In ones life when living for Christ, many believe that these kinds of conditions are brought on by demonic influences. Does anyone ever think to call upon the Lord God for deliverance in these cases?
44*	***Are there any alternative treatment options for schizophrenia?***
45*	Whenever i feel very disappointed because of failure, or humiliated, i feel like i should suicide and i start thinking of which is the easiest way to do so. Is it normal?
46	***What is the difference between Neurosis and Psychosis?***

47	***How does depression harm the brain?***
48	***Can borderline personality disorder be passed down genetically?***
49	Is it safe to marry an individual who is or was treated with mental disorders?. What is the chance in percentage that the children out of this relation will have the same disease?
50*	Do people who suffer from schizophrenia hear voices of people they know (or themselves) or are they voices that they don't recognize?
51	Do people with bipolar disorder or schizophrenia, enjoy more fulfilling romantic relationships with counterparts who also have these mental illnesses?
52	***Is depression hereditary?***
53	***Is recovery from depression based on a decision to be more positive?***
54	***What is the best natural remedy for Anxiety?***
55*	Is Bi-polar disorder curable at all or will people always be dependent on their medication?
56	What are the major side effects of selective serotonin reuptake inhbitors (SSRIs) which are used to treat depression?
57	***Can mental illness be caused by a poor diet?***
58	***Is Alzheimer's disease hereditary?***
59	***Is anxiety a form of depression? What triggers anxiety?***
60	***Can depression be associated with pregnancy?***
61	***What is the difference between bi-polar 1 and 2?***
62	Can people with down syndrome go to colleges or universities and succeed despite of their low IQ? Can they succeed at hard courses (like Calculus, Organic Chemistry, Microbiology...)?
63*	***What are the most common symptoms of anxiety?***
64	***How long does it typically take for Post Partum Depression to resolve itself?***
65	***What are some ways to conquer anxiety?***
66	***Can you recover from mental illness without the help of traditional medicine?***
67	***Why is depression more prevalent in women than in men? [repeated question]***
68*	if someone who went through a 2 week episode of major depressive disorder then is he/she likely to expereince that 2 week of depressive mode again?
69	***Do thoughts of suicide go away by them selves or do you need a doctors help?***
70	What causes depression, if anything? Does medicine really help against depression?
71	Is anxiety attributed more to genetics and "wiring" of the human brain, or conditions presented during developmental years?
72	I've always been told that everyone gets depressed or anxious sometimes in their life but when is the time to talk to a professional about it?
73	What type of life stressors can cause someone to experience depression? And what are the first signs of depression?
74	***At what age do symptoms of schizophrenia usually begin to show in those affected?***
75	***Can mental illnesses be cured by detoxing heavy metals?***

76	What are the natural ways of recovering from depression without using any medication?
77	***Does depression ever fully go away?***
78	***Suicide is most common with which mental illnesses?***
79	***How early do individuals with schizophrenia begin to manifest symptoms?***
80	***Are anxiety disorders and PTSD connected?***
81*	The brain is divided into multiple parts and the parts are responsible for certain functions that take place in the human body. Which part of the brain is associated with mental illness, depression etc. ?
82	What percentage of people who are diagnosed with Generalized Anxiety Disorder are able to control it and live a normal life?
83*	***Is depression linked to a certain gene since it does seem to run in families?***
84	Is depression caused by something that happens to a person in their past, or is it something the person is born with?
85	***Is depression manageable solely through talk therapy (with a psychiatrist)?***
86	***How do you know if your loved one has depression?***
87	Does the mental patients have a tendency to commit suicide and why? It is said that people who had multiple surgeries suffer mental illness. Is it true?
88	***Do you ever really cope with dealing with Depression in every day life?***
89*	***What internal coping mechanisms can you use to help deal with bi-polar disorder?***
90	***How do you treat OCD?***
91*	***How does depression in children under 10 years old effect them later in life?***
92	***Is post traumatic stress disorder permanent?***
93*	What is the best possible way that a person can recover from anxiety disorder, and is medication the best way to achieve such success?
94	Why do so many drugs on the market namely nerve medication cause depression or suicidial thoughts.
95	Are anxiety rates higher among university students compared to the general population?
96	Are mental illness hereditary?My father was a timid and nervous person so do myself.What to do to get my son free from this mental illness?
97	***Does the brain damage due to accident or illness make bipolar disorder?***
98	Do people ever recover from what I believe may be a rare mental ailment called Erotomania, and what may be the cause of this condition?
99	***Does schizophrenia only happen to people over a certain age?***
100	***Are people with schizophrenia violent?***
